# *Human Reproduction Open*, a wild mustang in the prairie of science

**DOI:** 10.1093/hropen/hoae022

**Published:** 2024-05-06

**Authors:** Edgardo Somigliana, Anja Pinborg, Andrew Williams

**Affiliations:** Editor-in-Chief, *Human Reproduction Open*; Deputy Editor, *Human Reproduction Open*; Managing Editor, ESHRE Journals

‘*What is Human Reproduction Open (HROpen)?*’ Since being appointed as the new editorial team of the journal, we have each been repeatedly asked this question. Our answer has always been the same, ‘*The authors will tell us*’. Journals are primarily shaped by their authors. And this is what occurred during this first year of activity. Perhaps surprisingly for such a young journal, we received and published many outstanding contributions, reflecting the high esteem that researchers hold for the journal. We were generally impressed by the utmost quality of the submitted manuscripts. Indeed, we have had to reject some seemingly high-quality studies, solely based on priority. Associate Editors and reviewers sometimes recommended the rejection of papers that, to our non-specialist eye, we found to be innovative and enlightening. The expertise and knowledge of our Editorial Board is second to none and their decisions unquestionable. We have unshakable faith in our Associate Editors. They are the heart of the journal and a guarantee of competence, quality, and pluralism.

This brings us to a second, more frequently asked, follow-on question: ‘*What’s the difference between Human Reproduction (HR) and HROpen?*’. At face value, this is potentially more difficult to answer, as both are clinical journals focusing on reproductive science and medicine. Where *Molecular Human Reproduction* and *Human Reproduction Update* have patent distinct characteristics, *HROpen* and *HR* appear not to. On the other hand, editorial activity is not mathematics. In many cases, *priority* guides the decision to publish or reject, but this is a poorly defined and subjective construct that widely differs among stakeholders. Fortunately, there is an ample spectrum of available journals for unsuccessful would-be *HROpen* authors to turn to. This is crucial to ensure a comprehensive acceptance of scientific contributions and to overcome unfair judgements on priority. If only one journal existed, we would run the risk of missing precious information, and the pace of science evolution would be slowed. For this reason, *HROpen* can exist, and peacefully coexist, with *HR*.

Finally, some historical perspectives should be considered. In fact, the vision for *HROpen* evolved over time. Initially, the journal was devised as a portal to capture the many good papers that were being rejected by *HR*, primarily due to priority. However, the first Editor-in-Chief, Siladitya Bhattacharya, had a slightly different vision. He mainly aimed at a journal that was ‘open’, in all senses of the term (Bhattacharya, 2017). This Open Access formula (fees paid to publish, not to read) allows the articles to reach everyone free of charge, including our target readership of researchers and physicians, regardless of their country of origin and economic availability. In the same way, science can also reach patients (the lay summary was introduced with the intent to offer patients comprehensible and accessible information that otherwise may be buried within the scientific and technical language of the article). ESHRE endorsed this view and moved the ‘ESHRE Pages’ papers (guidelines, recommendation, registries, reports of consortiums) that are of global interest, from *HR* to *HROpen*. However, the journal’s first Impact Factor was extraordinarily high (7.13—even above its elder sister), and the journal accelerated its own journey, independently, like a wild prairie mustang. The scenario changed. Submission sharply increased, and the quality astonishingly rose—even higher. The original notion that *HROpen* would simply ‘*capture the many good papers that were being rejected by HR*’ became something of a distant memory. As editors, rather than riding the mustang, we enjoy watching her running wild across the prairie.

We would like to express our immense gratitude to all the Associate Editors of the journal, the many wonderful reviewers and, last but not least, to all the authors who faithfully submitted their papers to our journal. Their outstanding contributions allow us to caress the muzzle of the mustang, gently and proudly.
